# Reproductive isolation in the acoustically divergent groups of tettigoniid, *Mecopoda elongata*

**DOI:** 10.1371/journal.pone.0188843

**Published:** 2017-11-28

**Authors:** Rochishnu Dutta, Tom Tregenza, Rohini Balakrishnan

**Affiliations:** 1 Centre for Ecology and Conservation, Department of Biosciences, College of Life and Environmental Sciences, University of Exeter, Penryn Campus, Penryn, United Kingdom; 2 Centre for Ecological Sciences, Indian Institute of Science, Bangalore, India; University of Arkansas, UNITED STATES

## Abstract

Sympatric divergent populations of the same species provide an opportunity to study the evolution and maintenance of reproductive isolation. Male mating calls are important in sexual selection in acoustically communicating species, and they also have the potential to maintain isolation among species or incipient species. We studied divergent south Indian populations of the bush cricket *Mecopoda elongata* which are extremely difficult to distinguish morphologically, but which exhibit striking divergence in male acoustic signals. We performed phonotactic experiments investigating the relative preference of females of the “Chirper” song type for calls of all 5 of the song types found in the region (in varying degrees of sympatry). We found that Chirper females preferred their own song type and were completely unresponsive to three trilling song types. Chirper females were occasionally attracted to the call type “Double Chirper” (the call most similar to their own type), suggesting call preference alone cannot provide a complete isolating mechanism. To investigate the basis of call preference we investigated the response of chirper females to variation in chirp rate. Chirper females responded most frequently to a mean chirp rate characteristic of their own song type rather than a higher chirp rate which would be more characteristic of the Double-Chirper song type. This suggests females drive stabilising selection on male chirp rate, which may contribute to the maintenance of isolation. Finally, a no-choice mating experiment using Chirper females and Chirper and Double Chirper males revealed a significant preference of Chirper females to mate with their own song type, even without a requirement for phonotaxis. Overall, the strong specificity of Chirper females for their ‘own’ song type provides evidence for behavioural isolation among divergent sympatric *Mecopoda* song types being maintained by female preference for both male song type and subsequent mating probability driven by other cues.

## Introduction

In many sympatrically distributed populations of acoustically communicating insects, divergent populations are morphologically indistinguishable (also termed cryptic) while differing considerably in calling songs that function to attract mates [1–3]. These acoustically divergent populations are often initially detected by their unique calls giving us a glimpse of the presence of unrealized diversity among many insect taxa (e.g. lacewings [4], ants [5], fruit flies [6], crickets and bush crickets [7–9], cicadas [10,11]). Acoustic signal divergence as simple as a change in pulse rate has the potential to create prezygotic isolation among populations [12–14]. Additionally Heller [15] and Shaw [16] have observed that different song patterns generally indicate distinct interbreeding populations that can be regarded as distinct species. As early as 1958, it was found that species-specific calls of otherwise morphologically similar grasshopper species of the genus *Chorthippus* acted as the main isolating barrier while other premating and postmating barriers seemed to have smaller effects [17]. The potential for auditory cues such as calling song to act as an agent of reproductive isolation has been identified in the sympatric Rotundifolia complex of the bush cricket *Amblycorypha* [8]. Evidence of reproductive isolation based on acoustic communication in bush crickets that are closely related and found in sympatry has also been identified in *Tettigonia* sp., *Neoconocephalus* sp. and *Acanthoplus* sp. [18–20].

*Mecopoda elongata* L. (Orthoptera, Phaneropteridae, Mecopodini) is a species of Asian bush cricket that has a number of song types (acoustically divergent populations) [21]. The males of five song types named Chirper, Double Chirper, Two Part, Helicopter, and Train, that are primarily recorded in southern peninsular India, are morphologically indistinguishable as are the females that lack the ability to call. These song types are distinct and non-overlapping in their temporal structure [22]. Populations of particular song types occur in different combinations of sympatry with one another. Although there are differences in mating season there are also considerable overlaps in the seasonal timing of calling and mating between sympatric populations [22]. The only two dyadic combinations (Chirper-Double Chirper and Chirper-Two Part) of this probable cryptic and incipient species complex are yet to be observed in sympatry although it is possible that these two are sympatric in some part of the range. Given the lack of morphological divergence, the obvious possibility is that co-inheritance of song type and female preference for song type means that gene flow between song type-populations is sufficiently low to allow them to retain their distinct identities. We investigate the arising prediction that females from a population with a particular song type will show a preference for males of that song type, both in terms of movement towards their calls and in terms of mating preference and we investigate the acoustic basis of any such preference.

Bush cricket males typically produce species-specific broadband calls to attract mates. Female bush crickets generally lack the ability to call, but respond to conspecific male calls by phonotaxis [15]. Our hypothesis is that in *Mecopoda*, females of a song type respond by phonotaxis to male calls from the same song type. The calls of *Mecopoda* play an important role in their reproductive biology as they allow females to locate, and potentially to choose among potential mates [23]. Field experience suggests that the *Mecopoda* are generalist in terms of food habit and no particular host plant preference is evident. The individual song type populations are also not associated with any particular habitat type. During field collections we found different song types thriving on similar shrubs and bushes within a small location. Males of more than one *Mecopoda* song type are often found calling simultaneously and interspersed with each other in their natural habitats. Although speciation by association to a limited host plant cannot be ruled out during an initial phase of isolation, it is unlikely to be a factor in maintenance of divergence among the song types at present. The considerable qualitative and quantitative differences in temporal features among the different song types make it very easy to identify which song type any particular male belongs to. There is no record of hybrid formation in the wild and the lack of intermediate song types suggests that hybridisation is rare [22], suggesting that speciation among the song types may be near completion. While other sexual signals such as cuticular hydrocarbon profiles may play a significant role in short distance communication, in this study we investigate the role of long distance communication in the maintenance of reproductive isolation. Male calls and female responses also serve as the first barrier to any mating occurring between *Mecopoda* song types. Since *Mecopoda* song types differ in temporal features of their calls while their spectral profiles are similar, the barrier to reproduction may lie among the temporal features of the song types as is common in many closely related bush crickets that use temporal features of their call for conspecific call recognition [19,20,24–26].

Among the five known song types of Indian *Mecopoda*, Chirper and Double Chirper calls consist of chirps only, while the other three song types (Two Part, Helicopter and Train) have trill components. Chirper calls are more closely similar to Double Chirper (having overlapping but lower chirp period) than to the other three trilling song types [22]. If the genetic integrity of Chirper populations is maintained by female choice based on acoustic cues alone, we expect Chirper females to prefer (show a tendency to move towards) the call of their ‘own’ type while ignoring calls of other song types. Such a pattern would indicate significant reproductive isolation between Chirper and the other song types. Therefore, our primary objective was to test the preference of *Mecopoda* Chirper females for their ‘own’ calls in comparison to their responses to other song types to examine isolation at the level of long distance acoustic signal (mating calls). Our second objective was to study post-contact mating behaviour to gain a more complete picture of the strength of behavioural isolation at the level of mating.

Many aspects of the temporal features of a cricket call play a role in female phonotaxis. For example, higher chirp rate is known to be a preferred trait among many cricket females [27–30] where calling effort is hypothesized to be a energetically expensive trait that may advertise male fitness [31,32]. This hypothesis predicts that Chirper females should exhibit preference for calls with higher chirp rate (lower chirp period) which may lead to erroneous phonotaxis to other song types that exhibit closely resembling calls. This may occur in case of Double Chirper calls that closely resemble the song type Chirper but feature two rapidly repeated chirps in place of a single chirp. In case Chirper females show preference to any of the other song types, our third objective was to identify the temporal features of *Mecopoda* calls contributing to the isolation of Chirper from other song type populations.

To study this, we first investigated the responses of Chirper females to a range of chirp periods of their own call type. This would allow us to determine whether Chirper females prefer lower chirp period (which may serve as an honest signal of male quality) or the mean chirp period of Chirper calls which would be more likely to maintain reproductive isolation from other *Mecopoda* song types. It has been argued that in fast-calling bush cricket species such as *Neoconocephalus* sp., females tend to ignore the fine scale doublet call structure and instead rely more on coarse features such as gaps or duration [33]. Therefore, there is a possibility that at higher chirp rate, Chirper females ignore the call structure of the faster calling Double Chirper and respond to it as they would to a low chirp period chirper male. If there is no significant preference for the other song types played out at Chirper females’ preferred rate, this indicates that temporal parameters other than chirp rate and chirp structure are responsible for Chirper females’ phonotactic decisions. This series of experiments allowed us to study the extent of behavioural isolation in Chirper song types of *Mecopoda* sp.

## Materials and methods

### Animal collection

**Fig 1:**
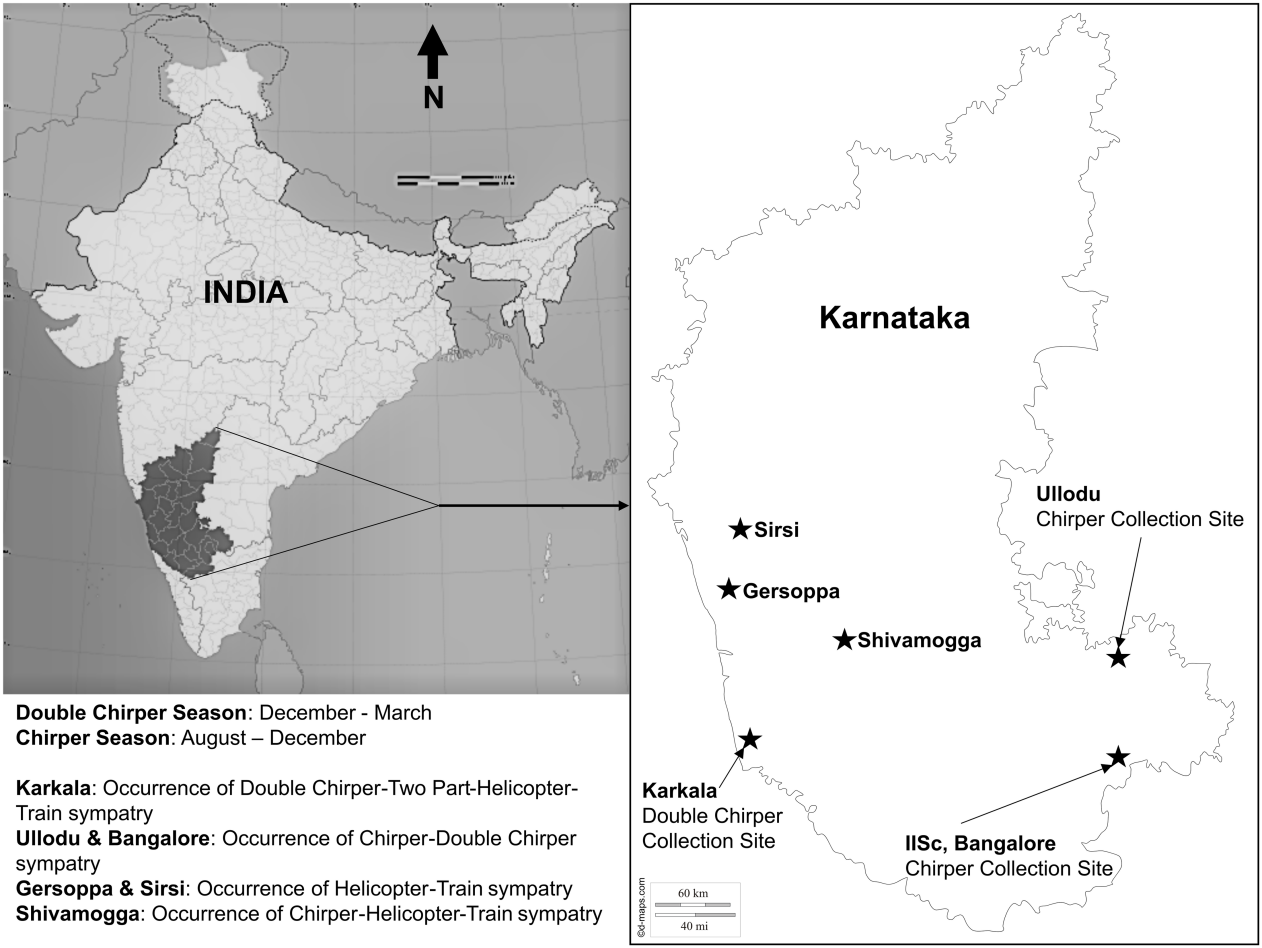
Map showing known occurrences of five *Mecopoda* song types in India. [22].

*Mecopoda* is univoltine, with eggs surviving the non-breeding period and contributing to the adult population of the next year. The mating season of the different song types varies but overlaps temporally [22]. A Chirper population of *Mecopoda elongata* is found on the campus [N13° 01’, E77° 34’] of the Indian Institute of Science, Bangalore, (IISc) that is otherwise devoid of co-occurring song types. Prior to this study, attempts to establish pure bred *Mecopoda* song types remained unsuccessful. However, we were able to establish a pure bred population of Chirper song type in culture for the first time in 2011 from the 2010 collection of 47 adult Chirper females that were collected from the campus. In 2011, 48 adult Chirper individuals, including 32 females, survived to adulthood (egg to adult turnover was approximately 8%). We bred these 32 females with new wild-caught Chirper males from the campus for the subsequent generation. To supplement the low population size of the laboratory bred females, some Chirper females used in the experiment were also caught as nymphs from the campus population and raised in culture to adulthood. Chirper males (from IISc campus) and Double Chirper males (from Karkala [N13° 13’, E75° 05’]) used in this study were mostly collected from already known sites [22]. Only two of the Chirper males used in the mating experiment were collected from a new site, Ullodu [N13° 38’, E77° 42’], 100 km from IISc campus (Fig 1). We failed to establish a pure bred culture of other song type populations for reasons that remain obscure, but presumably relate to something in the laboratory environment which failed to match their natural conditions. The culture maintenance of the other song type populations was unsustainable due to their relatively low fecundity (egg to adult survival was much lower than that of Chirper).

Nymphs were fed *ad libitum* on oat flakes (Quaker Oats, Morten Seeds & Grains Pty. Ltd.), fish food (Taiyo Grow, Taiyo Petproducts [P] Ltd.) and dog food (Pedigree, Mars International India Pvt. Ltd.) while calcium carbonate (Calcium Sandoz, Novartis India Limited) and cabbage leaves were given as supplements. We fed Chirper adults with fish food and oat flakes offered *ad libitum*. Water was also provided *ad libitum*. The culture room was maintained between 24°C and 30°C corresponding to the typical range of temperatures in their natural habitats. We maintained all Chirper females separately after they moulted into adults. They had no physical contact with any male before or during the experimental period. In the experiments, we used adult Chirper females that were at least 2 weeks beyond their final moult. Females used for experiments were acoustically separated from conspecifics on the experimental day and were placed in an anechoic room one hour before the commencement of experiments.

Ethics Statement: All protocols for behavioural data collection adhered to the national guidelines for the ethical treatment of animals laid out by the National Biodiversity Authority (Government of India). *Mecopoda* is not mentioned in both IUCN red list and Indian schedule species database. It is a widely distributed species in India and occur in both protected and non-protected areas near human habitation. All fieldwork during this study was conducted with the full consent and permission of the local stakeholders.

### Playback

The five different song types differ in their temporal features to an extent similar to that found between taxa at higher levels than species [22]. For playback, a basic unit of a call recording from an individual of each song type was chosen such that the song features approximated the mean of their distribution in natural populations. The chirp duration and chirp period of a representative playback call segment of each song type used in the acoustic playback are provided in the supplementary material (S1 Table). We collected segments of calls used in the phonotactic experiments from calls recorded previously using a Bruel and Kjaer Sound Level Meter 2231 mounted with a quarter-inch microphone (Bruel and Kjaer 4939, frequency range 4 Hz—70 kHz) [22]. The SPL for calls used in the playback at a distance of 1.7 m was 76.2 dB (peak), which is representative of SPLs under field conditions [34]. The mean background RMS sound pressure level was 52.3 dB ± 0.6 (Mean ± S.E.M). We looped the segments of each of the five songs through a MATLAB (The Mathworks Inc., Natick, MA) program to make a 10-minute call that we played from a laptop (MacBook Pro with OS X 10.9.4). We used an Avisoft power amplifier, a USB-sound card (NI USB-6215, National Instruments) and Avisoft speakers (Ultra-sound Scanspeak, frequency response: 1–120 kHz) to broadcast the calls.

### Experimental procedures

#### Experiment 1: Call preference of phonotactic Chirper females

The aim of this experiment was to study the phonotactic behaviour of Chirper females in response to playback of different *Mecopoda* song types. Since the number of females was limited, we followed a repeated measures design. All the phonotactic trials were completed within 5 hours of the onset of the dark cycle. The trials were run in an anechoic arena that was 2m x 2m in size. The two speakers were placed 150 cm from each other. The release point of the females was 170 cm from the speakers, the speakers and the release point forming an isosceles triangle. The mean temperature throughout the experimental period was 23.6°C ± 0.5°C. We tested each Chirper female over four days for response to Double Chirper, Two Part, Helicopter and Train calls with one stimulus trial per day. We also tested every female used in the experiment with Chirper calls (positive control) on each day. This was done to check for motivation of the females over different days as motivation varies among days as well as individuals. All trials were carried out between the 18^th^ and 34^th^ day post adult emergence. The order of presentation of animals (in the case of more than one female being tested on a day) and the choice of speaker (left or right) for a trial were randomized within each night. The playback order for each female was randomized over four nights. The total experimental time in a day was divided into two halves. In one half, we played one of the four other song types to the females. In the other half, the Chirper song type was played to the same female as a positive control. These halves were alternated over four days. If the female did not show any response to any call in a given experiment, the female was retested on a different night. All playbacks were 10 minutes long with 5 minutes for acclimatization given to the animals before each trial. During acclimatization, we held the female at the release point in the arena in a mesh cage (15 cm x 10 cm x 3 cm) that had the bottom side (15 cm x 10 cm) open. After 5 minutes, we began the call playback and removed the mesh cage (if females were free) or turned it upside down (if females clung onto the mesh). We recorded the exact time for phonotactic response (from release from mesh cage to reaching within 10 cm of the speaker). Only if a female showed response to the positive control was its phonotactic response used in the analysis. If a female responded to the positive control but not any other song type, the test was considered negative for the other song types, and if she responded to the positive control and any other song type then her response was considered positive for the other song type.

#### Experiment 2: Reproductive isolation based on mating between Chirper and Double Chirper

We carried out the mating experiment using a no-choice design since the probability of encounter in the wild is unknown. A no-choice design provides an overestimation of the mating probability since an encounter between mating individuals is ensured within a closed space [35]. We used a plastic box 23 cm x 13.5 cm x 10 cm with an open top (23 cm x 13.5 cm) covered with a transparent plastic sheet for the mating experiment. All Chirper females used in the trials were unmated adults. We chose males based on their calling status on the day before the commencement of the trials since calling serves as an indication of males’ eagerness to mate. An encounter (any physical contact) between the mating pairs occurred as soon as they were put in the box for all mating trials. We considered this to be the beginning of the trial. Males that did not call within 30 minutes after the female was introduced to the box were replaced with another male. The trials involving only Chirpers lasted for at least 1 hour while trials involving Double Chirper males were conducted for 2 hours with an assumption that Double Chirper males may take longer to initiate mating with a Chirper female. This experimental design will therefore inherently give a conservative (slight over-) estimate of between song type mating probability, but was chosen because our aim was to discover whether such inter-song type matings occur at any significant rate. We scored females as mated after a successful transfer of a spermatophore, visible at the base of the ovipositor of the females after mating. We also recorded the premating duration (duration from first encounter to the start of spermatophore transfer) and mating duration (time taken by a male to place spermatophore onto to the base of the ovipositor) of each mating event.

#### Experiment 3: Phonotactic preference of Chirper females towards different Chirper call chirp rates

We conducted this experiment to study Chirper females’ response to extreme chirp rates with respect to the mean chirp rate of Chirper calls and thus, to examine whether Chirper females are tuned to the mean chirp rate of their own call. We again followed a repeated measures design in order to make the most of the limited number of females we had available (total = 39 Chirper females). A similar experimental setup was used as in the study of call preference behaviour of Chirper females but only one speaker, instead of two, was used and it was placed facing the release point along a straight line. We tested each Chirper female over three different trials involving playback of mean (480 ms), mean + 2S.D. (755 ms) and mean– 2S.D. (210 ms) chirp period of Chirper calls over three consecutive days. The order of presentation of females (in case more than one female was tested on a given night) was randomised within a night. The playback order for each female was chosen randomly over three nights, as was the direction of playback from among East, West, South and North. At least 5 minutes was given to the females for acclimatization in the arena before each trial. Only if a female reached within 10 cm of the speaker was it scored as a positive phonotactic response.

#### Experiment 4: Call structure preference of phonotactic Chirper females

This experiment examined whether Chirper females show any significant preference to either Chirper or Double Chirper call structure when both calls were played back using Chirper females’ preferred chirp period (as found in the previous experiment) and when calls were played back using mean Double Chirper chirp period. We used the females from Experiment 3 following a repeated measures design in this experiment. The two phonotactic experiments consisted of two trials each, i.e., playback of Chirper and Double Chirper calls, at mean Chirper chirp period in one while the other consisted of the same playbacks at mean Double Chirper chirp period. We conducted each experiment within a given night. We alternated the order of playback such that the number of trials with Double Chirper playback conducted first was approximately equal to the number of trials with Chirper playback performed first. The experimental set up, procedure and criteria of scoring response used for this experiment were the same as in the previous experiment.

### Statistical analysis

All statistical analyses were carried out in R [36]. We used various statistical tests (Chi-square test, Fisher exact test, Cochran Q-test and McNemar’s test) depending on the nature of the dependent variables used to compare two or more explanatory variables. Since we used Chirper calls as a control in Experiment 1, the number of trials in which 21 Chirper females were subjected to Chirper calls was four times the number of trials that Chirper females were subjected to any of the other 3 call types. To make a statistical comparison of responses of Chirper females towards Chirper and Double Chirper calls (statistical analyses were not needed for the other 3 call types as females never responded to them), we used a resampling statistical approach using R. Our routine sampled 21 responses randomly out of all the trials that involved 21 Chirper females subjected to Chirper calls (including no responses and repeats). This process was reiterated 1000 times. Each time 21 randomly picked responses were generated and the number of positive responses to Chirper calls counted. We calculated the mean of these positive responses generated 1000 times and used the value as the true response of Chirper females towards Chirper calls. Pearson’s Chi-square test with Yates’ continuity correction was used for examining the response of the Chirper females to Chirper and Double Chirper calls. To compare the frequency of mating of Chirper females with different-song type and same-song type male, Monte Carlo simulation of p value using 10000 iterations was conducted to calculate a more robust p value. For the mating experiments, we performed Fisher exact tests to test the independence of the recorded response. In experiments 3 and 4, we followed Cochran’s Q test and McNemar’s test with continuity correction to check statistical significance of the differences in responses. Cochran’s Q test and McNemar test were used since the explanatory variables were not independent of one another due to repeated use of the same *Mecopoda* individuals in trials.

## Results

### Phonotactic Chirper females prefer ‘own’ calls but occasionally respond to Double Chirper calls

**Fig 2:**
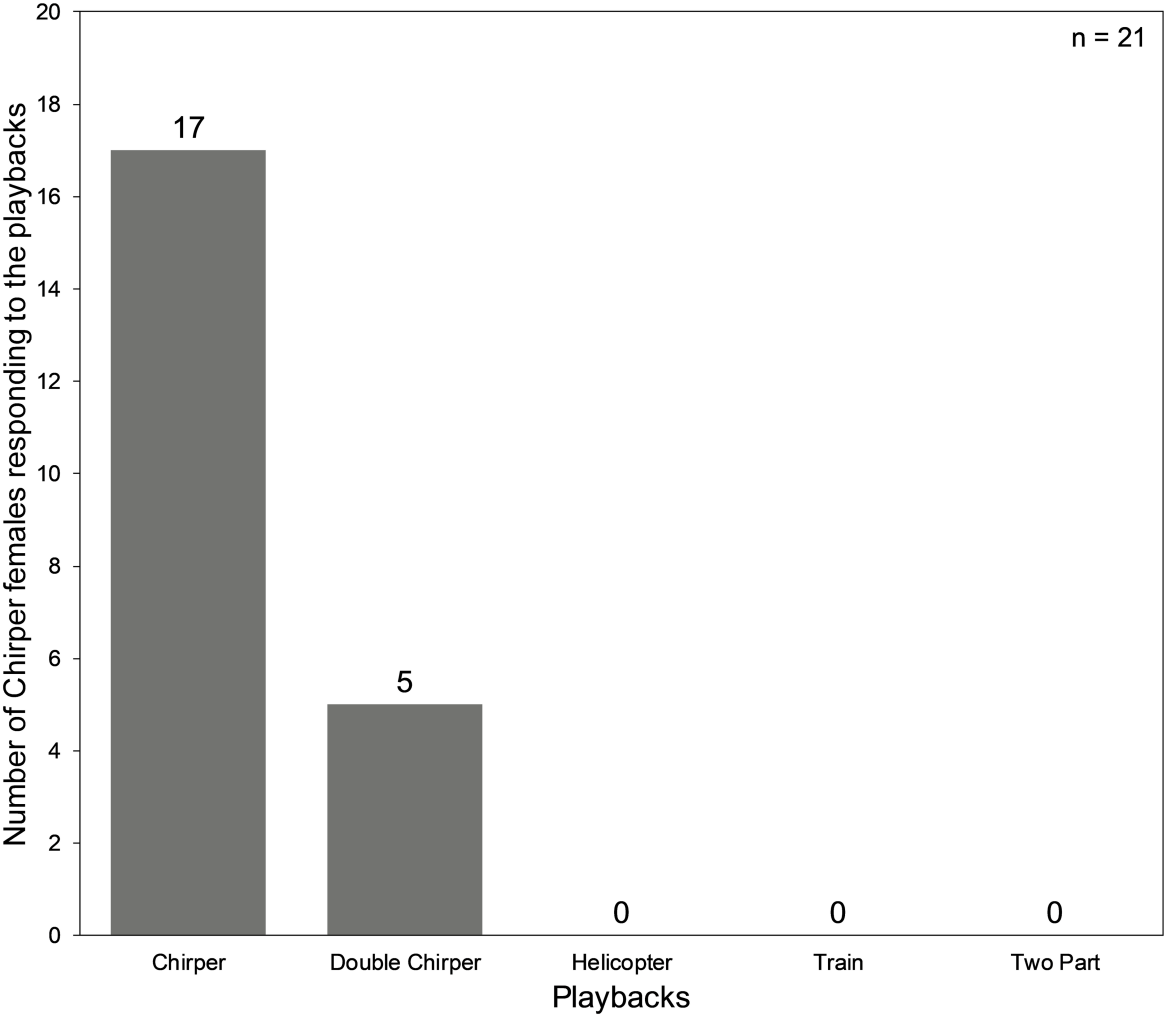
Phonotactic response by *Mecopoda* chirper females to the playback of calls from all *Mecopoda* song types.

Out of a total of 21 females that responded to the positive control (Chirper calls) on all four trials, 5 also responded to Double Chirper calls. None of the Chirper females showed any phonotactic response to any of the trilling song types (Two-Part, Helicopter and Train) in any trial (Fig 2). The mean frequency of phonotactic response of Chirper females towards Chirper calls from the statistical resampling method was 17 out of 21, a figure which was used in the following analysis. A Pearson’s Chi-square test showed a significant (Chi-square = 11.6, df = 1, p value = 0.00068) difference between response to Chirper calls and response to Double Chirper calls.

### Chirper females rarely mate with Double Chirper males

In the 12 mating trials involving Chirper males and females, 9 out of 12 females mated. Among the 8 trials where Double Chirper males and Chirper females were put together (this was difficult to achieve since mating season of these two song types were different with little overlap), only one instance of different-song type mating occurred. A Fisher exact test for count data with simulated p value (p = 0.0198) indicates that the difference in frequency of same-song type and different-song type mating by Chirper females is significant.

In all cases of same-song type mating, mating was preceded by brief Chirper calls sometimes continuing till the attachment of the male genitalia to that of the female and continuing calling bouts even after a successful mating. In the single case of different-song type mating, there was no calling by the Double Chirper male involved. From the same-song type mating experiments between Chirper males and females and one occurrence of different-song type mating, it was quite clear that there is no spermatophylax (a proteinaceous part of the spermatophore) offering by males in the *Mecopoda* mating. Frequently, male tettigoniids offer a spermatophylax to females during mating to prevent premature detachment of sperm containing ampulla [37]. None of the females removed the attached spermatophore or fed on them within the trial period. Although the mating usually occurred in less than 1 hour, the spermatophore was retained for much longer; in 5 of the Chirper females we observed spermatophore retention beyond their trial period. The mean time for which the spermatophore was retained was 5 ± 0.7 hours after the trial period, and spermatophore retention time was never more than 12 hours.

### Phonotactic Chirper females prefer the mean chirp period of their ‘own’ call

**Fig 3:**
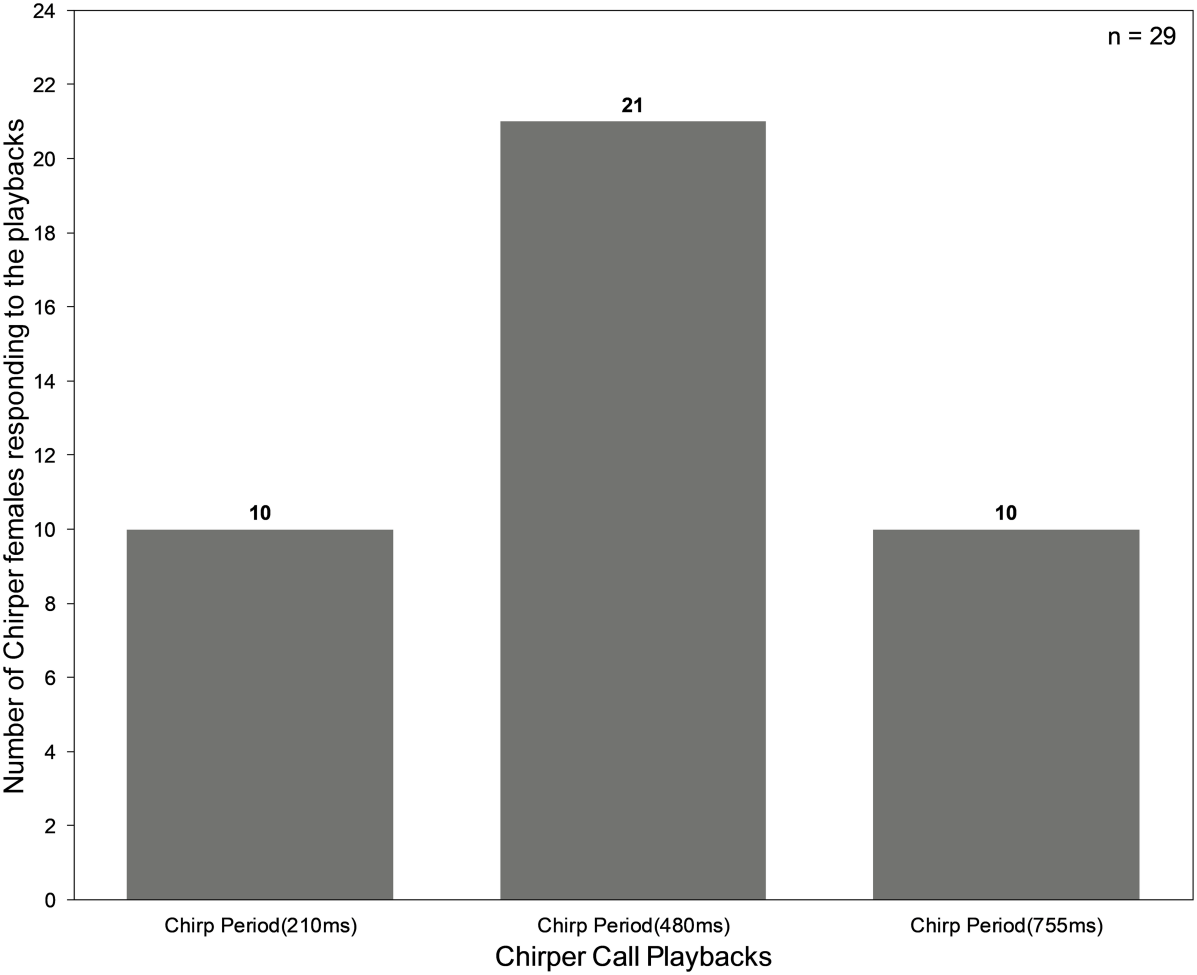
Phonotactic response by *Mecopoda* Chirper females to Chirper calls played at mean, mean + 2S.D. and mean- 2S.D. chirp periods.

Out of 29 Chirper females that responded in at least one of the trials, 21 responded to the mean Chirper call (480ms), 10 each responded to mean + 2S.D. and mean - 2S.D. Chirper chirp periods (Fig 3). The frequency of responses to the 3 chirp periods were significantly different from each other (Cochran’s Q test, Q = 8.64 and p = 0.0133).

### Phonotactic Chirper females prefer the chirp structure of their ‘own’ call

Out of 27 Chirper females that responded to at least one of the trials, only 4 responded to Double Chirper calls played at mean Chirper chirp period (480ms) while 26 females responded to the Chirper call played at mean Chirper chirp period (480ms). There was a significant difference between the responses to the two treatments (McNemar Chi-square test with continuity correction: G = 18.4, p = 0.000018, degrees of freedom = 1). Similarly, out of 27 Chirper females that responded to at least one of the trials, 24 responded to Chirper calls played at mean Double Chirper doublet period (380 ms) while 9 females responded to the Double Chirper call played at mean Double Chirper chirp period (380 ms). There was a significant difference between the responses to the two treatments (McNemar Chi-square test with continuity correction: G = 9.33, p = 0.00225, degrees of freedom = 1).

**Fig 4:**
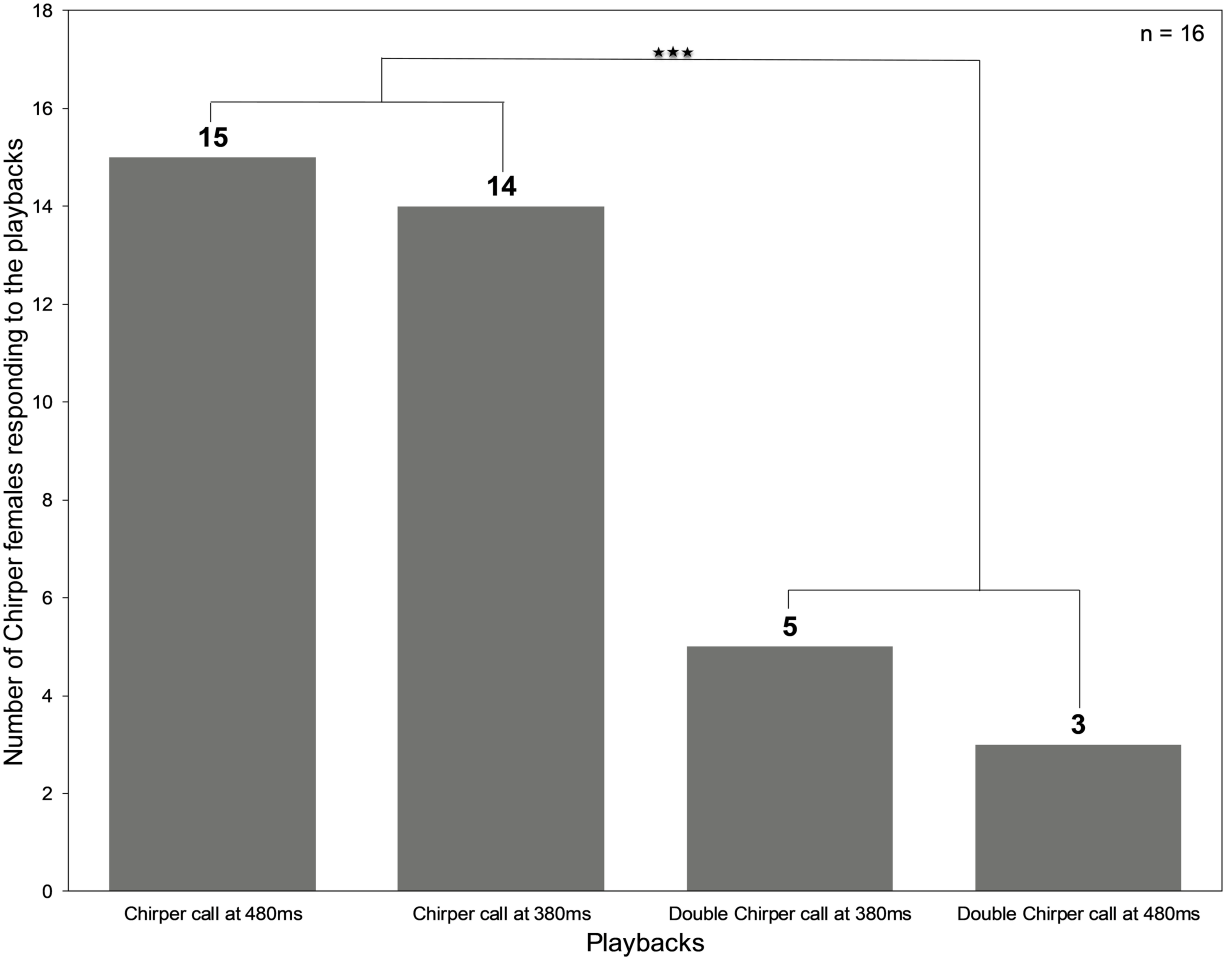
Phonotactic response of *Mecopoda* Chirper females to Chirper calls played at chirp periods of 480 ms and 380 ms and Double Chirper calls played at chirp periods of 480 ms and 380 ms.

When considering 16 Chirper females that responded to trials in both the above mentioned experiments, 15 females responded to mean Chirper calls, 14 females responded to Chirper call played at Double Chirper doublet rate, 5 females responded to mean Double Chirper calls and 3 females responded to Double Chirper calls played at mean Chirper rate (Fig 4). A Cochran Q-test (Q = 21 and d.f. = 3, p = 0.00011) indicated that the overall probability of a female response was significantly greater in response to chirper than to double chirper calls.

## Discussion

### Premating isolation among the song types of *Mecopoda*

Reproductive behavior in orthopterans typically includes female phonotaxis to a calling male, antennation to confirm species level specificity that ultimately concludes in copulation between the mating pair [15]. A successful phonotaxis reveals that the female of a cricket has recognised the call of a conspecific male and could localize it [18]. The absence of a response to Two-Part, Helicopter and Train playbacks in phonotaxis experiments indicates that Chirper females strongly discriminate against calls of these three song types. This is almost certain to create strong premating isolation between populations of these three song types and Chirper. Given that the spectral profiles of all the *Mecopoda* song types are relatively similar, it also indicates that temporal feature divergence is responsible for the observed premating isolation as observed in the studies of speciation in other bush crickets [8,19,20,24–26]. One common feature among the three song types that were completely discriminated against by Chirper females is that their calls include a trill component, suggesting that this aspect of the song is important in discrimination.

Chirper females showed a significant preference for Chirper calls over Double Chirper calls. The occasional phonotaxis of Chirper females towards Double Chirper calls however indicates incomplete premating isolation via phonotaxis between populations of these two song types. This could be because Chirper and the Double Chirper calls are more similar to each other, with overlapping distribution of their temporal features and both lacking any trill component. This similarity in calls may have led a proportion of Chirper females to respond to the calls of Double Chirper. It may also be that the incomplete premating isolation between Chirper and Double Chirper is due to lack of any selection pressure to avoid hybridization since there is no evidence of their geographical co-occurrence. There is a general trend of existence of stronger discriminating mechanisms among the divergent groups of a species found in sympatry than the divergent populations of a species that are found in allopatry [38].

Reproductive isolation between Chirper and Double Chirper was further manifested at the level of mating between individuals of the two song types. The expected low probability of a successful mating between Chirper and Double Chirper was confirmed by our mating experiment in which individuals were placed in close proximity. The single instance of a Double Chirper male mating with a Chirper female may represent an anomalous event that will only occur when animals have a no-choice situation [35]. Alternatively, this mating could be interpreted as an evidence of incomplete reproductive isolation at the level of mating. However, if we take the probability of mating between Chirper females and Double Chirper males in our study (0.125) and the probability of Chirper females showing phonotaxis towards Double Chirper males (which is 0.24) we get an even lower probability of 0.03 with which Chirper females actually mate with Double Chirper males after a successful phonotaxis. This estimate ignores the fact that the confined proximity of mating pairs in our mating experiment may lead to an overestimate of mating probability. It is therefore safe to assume that the chance of mating between Chirper and Double Chirper is low although the two song type populations do appear to have the potential to mate if they were to occur in sympatry. Whether or not such matings will lead to successful production of hybrid offspring is unknown, and given the difficulties of rearing these animals in captivity, may remain so. Reproductive isolation may also be promoted by other sexual cues such as cuticular lipid profiles that *Mecopoda* may use to discriminate among mates during antennation. Preference for certain cuticular lipid profiles can further prevent individuals from two different song types from eventual mating in case song preference fail to deter some individuals inform coming into close contact. This may certainly happen in case the distribution of Chirpers and Double Chirpers overlaps. Nevertheless, the ability of acoustic signals to maintain isolation as a first line of reproductive barrier is beyond doubt. Our study provides persuasive evidence of reproductive isolation being maintained through behavioural isolation between five incipient species of the *Mecopoda* genus.

### Acoustic traits involved in behavioural isolation between Chirper and Double Chirper

The temporal features of bush cricket calls such as pulse rate, duty cycle, pulse duration and the duration of gaps are used singly or in combination by many species for conspecific recognition and heterospecific discrimination [19]. Given this, there are two possible proximate explanations for Chirper females occasionally showing phonotactic behaviour towards Double Chirper calls. Firstly, Chirper females might be influenced by the higher chirp rate of the Double Chirper call in relation to the Chirper call since higher chirp rate is generally a preferred trait [31,32] especially when they are energy intensive and form the basis of female choice. If chirp rate in Chirper is under directional selection within the song type, with females preferring higher same-song type chirp rate, this could provide one possible explanation for approach towards Double Chirper calls, which are produced at higher rates. The experiment examining the preferences of Chirper females for chirp rates of their own call type (spanning the natural distribution of Chirper male calls) however indicates stabilizing rather than directional selection on chirp rate. The preference for chirp rate at both ends of the chirp rate distribution is also symmetrical (Fig 3). This is not surprising because temporal features that primarily serve as species recognition signals instead of traits under mate choice are thought to be highly conserved and help in maintaining species identity [32]. Therefore, Chirper female preference for mean chirp period may be acting as the basis of assortative mating between Chirper individuals in case disruptive selection has previously isolated the two song type populations. Thus, a preference for higher chirp rates driven by directional selection on lower chirp period is unlikely to explain the occasional phonotaxis of Chirper females towards Double Chirper calls.

Alternatively, some Chirper females might be less sensitive to the call structure in general and hence orient to both Chirper and Double Chirper calls. Chirper females may evaluate Double Chirper doublet structure as a contiguous call rather than a doublet based on the total call duration as the distribution of doublet duration of Double Chirper (117 ms to 236 ms) overlaps considerably (about 45 ms) with the distribution of chirp duration of Chirper calls (56 ms to 162 ms) [22]. This possible error might be because in species where males call at higher chirp rate, females generally depend on gross structural cues (such as gaps or overall chirp duration) for species recognition [33]. This study suggests that most Chirper females discriminate against the temporal feature of the Double Chirper call (Fig 2) although some also prefer the suboptimal temporal features of Double Chirper. Even when Double Chirper call is played at the preferred chirp rate of 480 ms, Chirper females do not show any significant increase in the response to Double Chirper calls but have strong preference for their own calls. This indicates that Chirper females pay attention to the call structure. This difference in preference for playbacks is also maintained when Chirper calls are played at the mean Double Chirper chirp rate of 380 ms. The most likely explanation for some Chirper females orienting towards Double Chirper call is, thus, a weaker preference for temporal parameters of the Chirper call other than the chirp rate and chirp structure. Future work to determine the exact call trait(s) responsible for the precise phonotactic behaviour of Chirper females will require more sophisticated manipulation of call components.

## Conclusion

Our study suggests that there is a complete behavioural isolation between Chirper individuals and individuals from the three trilling song types, namely, Two-Part, Train and Helicopter at the level of phonotaxis, which is important in forming mating pairs. The possibility of matings in the wild between Chirper and these song types seems highly unlikely. A near complete behavioural isolation between Chirper and Double Chirper exists but with a possibility of gene exchange when phonotaxis, mating and known distribution of these two song types are considered. Chirper females show occasional phonotaxis to Double Chirper calls that cannot be attributed to the preference of Chirper females to chirp rate and chirp structure of the Double chirper calls. Rather this is highly unlikely in the wild due to their non-overlapping distribution. The likely explanation seems to be weak attraction of Chirper females for some Double Chirper temporal call parameters other than chirp rate and chirp structure. However the overall picture indicates that Chirper song types may be considered as a separate cryptic species of *Mecopoda elongata* that has completed speciation and is maintained in reproductive isolation through divergence in acoustic communication.

## Supporting information

S1 Table Call duration and the call period of the selected call segments for playback of each *Mecopoda* song types. (DOCX)
