# Supplementary material for: Reproductive isolation in the acoustically divergent groups of tettigoniid, Mecopoda elongata
Source: PLoS One. 2017 Nov 28;12(11):e0188843. doi: 10.1371/journal.pone.0188843 (PMC5705119; doi:10.1371/journal.pone.0188843)
Supplement: S1 Table — (DOCX) [file pone.0188843.s001.docx]

**Supporting information**

**S1 Table. Call duration and the call period of the selected call segments for playback of each *Mecopoda* song types**

| **Song types** | **Call Duration** | **Call Period** |
| --- | --- | --- |
| Chirper | 115 ms | 0.48 s |
| Double Chirper | 174 ms | 0.38 s |
| Two Part | 1.8 s | 1.9 s |
| Helicopter | 36 s | 36 s |
| Train | 96 s | 96 s |
